# Developing strategies to optimize the anchorage between electrospun nanofibers and hydrogels for multi-layered plasmonic biomaterials†

**DOI:** 10.1039/d3na01022h

**Published:** 2024-01-30

**Authors:** Yasamin Ziai, Massimiliano Lanzi, Chiara Rinoldi, Seyed Shahrooz Zargarian, Anna Zakrzewska, Alicja Kosik-Kozioł, Paweł Nakielski, Filippo Pierini

**Affiliations:** a Department of Biosystems and Soft Matter, Institute of Fundamental Technological Research, Polish Academy of Sciences Warsaw 02-106 Poland fpierini@ippt.pan.pl; b Department of Industrial Chemistry, University of Bologna 40136 Bologna Italy

## Abstract

Polycaprolactone (PCL), a recognized biopolymer, has emerged as a prominent choice for diverse biomedical endeavors due to its good mechanical properties, exceptional biocompatibility, and tunable properties. These attributes render PCL a suitable alternative biomaterial to use in biofabrication, especially the electrospinning technique, facilitating the production of nanofibers with varied dimensions and functionalities. However, the inherent hydrophobicity of PCL nanofibers can pose limitations. Conversely, acrylamide-based hydrogels, characterized by their interconnected porosity, significant water retention, and responsive behavior, present an ideal matrix for numerous biomedical applications. By merging these two materials, one can harness their collective strengths while potentially mitigating individual limitations. A robust interface and effective anchorage during the composite fabrication are pivotal for the optimal performance of the nanoplatforms. Nanoplatforms are subject to varying degrees of tension and physical alterations depending on their specific applications. This is particularly pertinent in the case of layered nanostructures, which require careful consideration to maintain structural stability and functional integrity in their intended applications. In this study, we delve into the influence of the fiber dimensions, orientation and surface modifications of the nanofibrous layer and the hydrogel layer's crosslinking density on their intralayer interface to determine the optimal approach. Comprehensive mechanical pull-out tests offer insights into the interfacial adhesion and anchorage between the layers. Notably, plasma treatment of the hydrophobic nanofibers and the stiffness of the hydrogel layer significantly enhance the mechanical effort required for fiber extraction from the hydrogels, indicating improved anchorage. Furthermore, biocompatibility assessments confirm the potential biomedical applications of the proposed nanoplatforms.

## Abstract 2

Interface in multi-layer plasmonic hydrogel/fiber nanocomposites supports a stable platform. Impact of various properties of the nanofibrous layer, and plasmonic hydrogel layer on the intralayer adhesion, and their biocompatibility was investigated.

## Graphical abstract

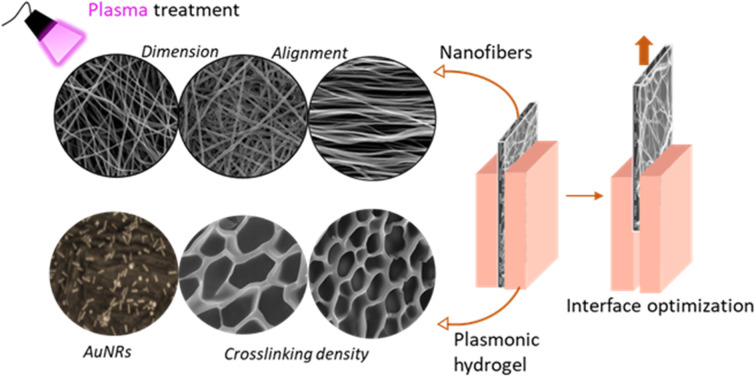

## Introduction

Recent years have witnessed remarkable progress in the field of biomedical materials, leading to the development of structures and platforms tailored for specific applications. These advancements aimed to harness the unique capabilities of various materials while addressing their inherent limitations. A particularly promising approach involves using composite materials, which combine the strengths of multiple materials within a single platform.^1,2^ Among these composites, hydrogel/fiber combinations have garnered substantial attention because they offer the potential to synergize the benefits of hydrogels and fibers while mitigating their respective shortcomings.^3,4^

Hydrogels, characterized by their three-dimensional structures with significant water content, biocompatibility, and tuneable mechanical characterizations, are particularly suitable for various biomedical applications,^5^ including drug delivery,^6^ tissue engineering,^7^ wound healing,^8^*etc.* One of the most significant advantages of hydrogels is their resemblance to the natural extracellular matrix (ECM), which allows cells to proliferate, differentiate, and migrate within these structures. This ECM-mimicking property is particularly beneficial in tissue engineering, where hydrogels can provide a supportive environment for cells to grow and form new tissues.^9,10^ Due to hydrogels' interconnected porous structure, they have been considered to be one of the best substrates to carry plasmonic nanoparticles for different applications. Due to their unique optical properties arising from interactions with incident light, plasmonic nanoparticles have been extensively explored in various fields. Noble metals, notably gold (Au) and silver (Ag) which are recognized as the most significant plasmonic materials, are characterized by the occurrence of surface plasmon resonance (SPR) at distinct resonance frequencies within both the visible and near-infrared (NIR) spectra.^11^ When these metals are utilized in the form of nanoparticles, a phenomenon known as localized surface plasmon resonance (LSPR) is observed. This LSPR can be effectively tuned by manipulating the size and shape of the nanoparticles to suit specific applications. Such tunability offers versatility in customizing their optical properties, thereby expanding their applicability in various applications.^12^

When incorporated into hydrogels, these nanoparticles can leverage their solid and tunable optical responses, enabling the manipulation of photothermal effects upon external light exposure. This combination has been particularly advantageous for biomedical applications such as biosensing^12^ and photothermal therapy.^13^ The engagement of hydrogels and nanoparticles has led to the development of advanced nanocomposite hybrid platforms, which benefit from both hydrogels and nanoparticles, offering stimuli-responsive features and expanding their range of applications.^14,15^ Plasmonic hydrogels can offer precise biosensing platforms, with low detection limits due to their biorecognition efficiency.^16^ Upon exposure to near-infrared (NIR) light, these nanoparticles can rapidly heat up, leading to a localized temperature rise. Combined with biocompatible and flexible hydrogels, these materials can be used in a wide range of applications such as photothermal therapy,^17^ targeted drug delivery systems,^18^ wound dressing,^19^*etc.* However, as the majority of materials, hydrogels present some limitations. One of the primary shortcomings of hydrogels is their relatively low mechanical properties in terms of modulus. While some hydrogels can be engineered for specific mechanical strengths, many naturally derived hydrogels lack the robustness and durability required for certain bio-applications, especially in load-bearing tissues like cartilage or bone.^20^ One of the effective procedures to reinforce hydrogels from a mechanical point of view is proposed to be the integration of fibers into a hydrogel network.^21,22^

The electrospinning technique is a well-established method for fabricating fibrous biomaterials and has been widely studied by numerous research groups.^23^ This process involves the use of a high-voltage power supply to create nanofibers with a broad range of diameters, from the micro-to nanoscale. Researchers can optimize various processing parameters, such as voltage, working distance, needle size, and flow rate, to tailor the material's properties to specific needs.^24^ Nanofibers have found applications in various biomedical fields, including tissue engineering,^25,26^ drug delivery,^27^ cell carriers,^28^ wound healing,^29^ biosensing,^30^*etc.* They offer a range of advantageous features, including a large surface area, tuneable porosity, a possibility for easy surface functionalization, and sufficient mechanical properties. One of the notable advantages of nanofibers is their ability to provide an ideal biocompatible substrate for culturing many types of cells. This is due to their high interconnected porosity, gas permeability, and capacity for fluid absorption.^31^ However, challenges arise regarding the infiltration of cells within the matrix, mainly due to the two-dimensional nature of electrospun mats, where there is a lack of cell-recognition sites especially in synthetic polymers.

Moreover, tissues with lower mechanical strength, such as brain tissue, pose unique challenges that electrospun fibers alone may not adequately address.^32^ Incorporating nanofibers into hydrogels has been investigated widely due to the reinforcement and enhancements the nanofibers introduce in terms of mechanical properties. There are several techniques to add these components, although the layering technique, where the two layer are in direct contact, can remodel the structures of tissues and develop adaptable platforms for different required load bearings.^33^

Hydrogel/fiber composites represent a compelling fusion of electrospun nanofibers and hydrogels, offering a platform that benefits from both materials' strengths while overcoming their weaknesses. Nakielski *et al.* have introduced a layered nanocomposite inspired by the natural structure of mesoglea of jellyfish bells for drug delivery purposes. This layered structure consists of two layers of electrospun poly(l-lactide) nanofibers loaded with Rhodamine B, and a layer of poly(*N*-isopropylacrylamide) (PNIPAAm)-based hydrogel with plasmonic gold nanorods. The hydrogel layer has been placed between two layers of nanofibers, allowing for controlled, rapid, and reversible structural changes upon NIR light irradiation. The mechanical contraction of the composite, triggered by temperature increases from plasmonic hydrogel–light interactions, can lead to rapid water expulsion, showing the importance of having a solid interface keeping the platform in shape while changing. This action, in conjunction with the temperature rise, stimulates the release of molecules from the nanofibers, making it an efficient platform for controlled drug delivery.^34^ In another work, Mohabatpour *et al.*, have developed a novel nanocomposite hydrogel scaffold by incorporating electrospun PLA (poly-lactic acid) nanofibers within alginate-grafted-hyaluronate (Alg-*g*-HA) hydrogel. The hydrogel scaffold demonstrated enhanced mechanical properties and reduced water uptake due to the embedded PLA nanofibers. Furthermore, the scaffold exhibited cytocompatibility, with chondrocytes maintaining their morphology and producing cartilage-specific matrix components, suggesting its potential application in cartilage tissue engineering.^35^

In this study, we have investigated a crucial aspect of these composites: ensuring a robust attachment between the fibrous mats and plasmonic hydrogel layers. In this work, polycaprolactone (PCL) was chosen as a representative biopolymer with a wide range of bio-applications. The study explores the influence of fibers' dimensions, alignment, and surface modifications on the fabricated fibrous mats' wettability, structural, and mechanical aspects. Polyacrylamide-based hydrogels with the incorporation of plasmonic gold nanorods have been introduced as the hydrogel layer. From this point of view, the effect of crosslinking density on integrating two layers was studied.

Additionally, biocompatibility tests were conducted on the developed platforms to assess the platforms' potential for biomedical applications. This research contributes to the evolving hydrogel/fiber composites field, offering valuable insights into multi-layer biofabrication. Structural, chemical, morphological, and mechanical enhancements in each layer and their impact on having a strong interface have been investigated.

## Materials and methods

### Materials

To prepare the nanoplatforms, polycaprolactone (PCL, Mn 80 kDa), *N*,*N*-dimethylformamide (DMF), chloroform (99%), *N*,*N*-isopropylacrylamide (NIPAAm, 97%), *N*-isopropylomethacrylamide (NIPMAAm, 97%), *N*,*N*′-methylene bisacrylamide (BIS-AAm, 99.5%), 2-hydroxy-4′-(2-hydroxyethoxy)-2-methylpropiophenone (Irgacure 2959, 98%), ammonium persulfate (APS, 98%), *N*,*N*,*N*′,*N*′-tetramethylethylenediamine (TEMED, 99%) were bought from Sigma-Aldrich (Poland). Gold nanorods (AuNRs, *λ* = 800 nm, OD = 50, *C* = 0.88 mg mL^−1^) from nanoComposix (USA) were used as received.

To perform biocompatibility tests, L929 murine fibroblasts, bovine serum albumin (BSA), phosphate buffer saline (PBS), Triton X, and DAPI were obtained from Sigma-Aldrich (Poland). Dulbecco's modified Eagle's medium (DMEM), fetal bovine serum (FBS), penicillin-streptomycin (PS), and EDTA-trypsin were bought from Gibco Invitrogen (USA). Alexa Fluor 488 Phalloidin, PrestoBlue reagent and live/dead cytotoxicity kit assay were purchased from Thermo-Fisher Scientific (USA).

### Electrospinning

Solutions containing PCL at concentrations of 10% and 14% w/v were prepared in a solvent mixture comprising chloroform and DMF (9 : 1) for the electrospinning process. Through a series of optimizations, nanofibers were successfully generated, with key electrospinning parameters set as follows: a flow rate of 0.5 mL h^−1^, employment of a 26 G needle featuring an outer diameter of 0.45 mm, and an applied voltage of 12.5 kV. In order to collect the resulting nanofibers in either a random or aligned configuration, a flat collector and a drum collector with a rotating speed of 2000 rpm were employed, positioned at a working distance of 15 cm from the electrospinning needle, respectively. Temperature of 20 °C, 40% relative humidity, and these optimized parameters were thoughtfully chosen to yield the desired nanofibers' morphology and alignment while ensuring the effectiveness of the electrospinning process. Nanofibers with 10% aligned (PCL 10A) and random orientation (PCL 10R) and 14% aligned (PCL 14A) and random orientation (PCL 14R) were fabricated and used for further studies.

### Surface functionalization

Diener Zepto oxygen plasma generator machine was used for surface modification of the nanofibrous layer. Nanofibers were cut in the desired shapes as rectangles and placed in the chamber for plasma treatment with a generator with a frequency of 40 MHz and power of 100 W for 2 minutes.

### Preparation of the hydrogel precursor solution

For the formulation of the hydrogel precursor solution with a concentration of 4.8 wt%, a precise combination of components was executed. This included the addition of 578.1 mg of NIPAAm, 15.6 mg of NIPMAAm, 31.2 mg of BIS-AAm, and 12.5 mg of Irgacure 2959. This composition was introduced into 10 mL of deionized water. This solution was used to prepare hydrogels *via* photo-polymerization (Ph-P).

0.1 wt% APS solution and 0.04% v/v of TEMED were added to the precursor solution to add the effect of chemical polymerization (Ph-P + C-P). To safeguard the solution from the effects of light, the mixture was securely enveloped in aluminum foil and subjected to continuous stirring overnight until complete dissolution was achieved.

This careful preparation ensured the uniformity and stability of the hydrogel precursor solution, setting the stage for subsequent experimental procedures.

### Fabrication of the nanoplatforms

Nanofibers were carefully tailored to the 3 × 0.5 cm × cm and were positioned into a mould with the dimension of 4 × 1 cm × cm. A 2% w/v solution of gold nanorods was introduced into each hydrogel precursor solution before the polymerization process. To facilitate photo polymerization, argon gas was introduced into the solution for a duration of 10 minutes, effectively displacing oxygen. 200 μl of each type of hydrogel precursor solution was then gently added over the nanofibers within the mould. The mould was carefully immersed in an ice bath to maintain the temperature at a controlled level below 15 °C during the UV irradiation process. Following this step, the mould, was exposed to UV light (Dymax lamp with 400 W capacity and a power density of 225 mW cm^−2^). The exposure time to UV irradiation was precisely adjusted, varying from 60 to 120 seconds, contingent upon the quantity of hydrogel material present.

Table 1 shows the list of the materials tested, with their acronyms.

**Table 1:** Samples description and acronyms

	PCL concentration (%)	Nanofibers orientation	Plasma surface treatment	Ph-P hydrogel	Ph-P + C-P hydrogel
10A/Ph-P	10	Aligned	No	Yes	No
10R/Ph-P	10	Random	No	Yes	No
14A/Ph-P	14	Aligned	No	Yes	No
14R/Ph-P	14	Random	No	Yes	No
10A + plasma/Ph-P	10	Aligned	Yes	Yes	No
10R + plasma/Ph-P	10	Random	Yes	Yes	No
14A + plasma/Ph-P	14	Aligned	Yes	Yes	No
14R + plasma/Ph-P	14	Random	Yes	Yes	No
10A/Ph-P + C-P	10	Aligned	No	Yes	Yes
10R/Ph-P + C-P	10	Random	No	Yes	Yes
14A/Ph-P + C-P	14	Aligned	No	Yes	Yes
14R/Ph-P + C-P	14	Random	No	Yes	Yes
10A + plasma/Ph-P + C-P	10	Aligned	Yes	Yes	Yes
10R + plasma/Ph-P + C-P	10	Random	Yes	Yes	Yes
14A + plasma/Ph-P + C-P	14	Aligned	Yes	Yes	Yes
14R + plasma/Ph-P + C-P	14	Random	Yes	Yes	Yes

### Chemical and morphological analysis

Contact angle measurements were done using an OCA 15EC goniometer. Droplets of 1 μl were placed on the surface of the PCL nanofibers, and the contact angle of 10 drops was measured and averaged using ImageJ.

Fourier transform infrared (FT-IR) spectroscopy was used to characterize the functional groups in each layer. FT-IR analyses were conducted in attenuated total reflectance (ATR) mode with a Bruker Vertex70 FT-IR spectrometer and carried out in the wavenumber range of 400–4000 cm^−1^ with a resolution of 2 cm^−1^ and eight scans for each sample.

Molecular weights were determined at room temperature by gel permeation chromatography (GPC) using CHCl_3_ solutions on an Agilent PL-GPC 50 apparatus equipped with a mixed bed column Waters Styragel HR 4E at a 1 mL min^−1^ flow rate.

Field emission scanning electron microscopy (FE-SEM) and scanning electron microscopy (SEM) were performed with FEI Nova NanoSEM 450 and JEOL JSM-6390LV microscopes, respectively. Hydrogel disks and layered constructs were frozen in liquid nitrogen before cross-sectional cutting and then freeze-dried. Before imaging, samples were sputtered with an approximately 8 nm thick gold layer using a SC7620 Polaron mini sputter coater (Quorum Technologies Ltd, Ashford, UK).

Fiber dimension distribution analysis was done using ImageJ software, investigating 50 fiber diameters for each condition. The same software was used to measure the contact angle of the droplets, examining ten droplets per condition.

### Mechanical tests

Both plasma-treated and untreated PCL specimens were tested to measure tensile strength. Rectangular samples of electrospun nanofibers, measuring 4.0 cm by 1.0 cm, were crafted for this purpose. These samples were secured within the clamps of the tensile testing device using a CTX Texture Analyzer (Brookfield Ametek), setting a gauge length of 10 mm. Data acquisition occurred at a rate of 50 readings per second. The actual testing commenced when the load reached 0.1 N. Samples were stretched at a rate of 1 mm s^−1^ until rupture in triplicates for each scenario; force–displacement graphs were plotted using the Texture Pro V1.0 Build 19 software. The results were plotted as stress–strain graphs, considering stress as the force-to-initial area ratio and strain as the relative change in sample length. Subsequently, Young's modulus was determined for each specimen, representing the stress-to-strain ratio in the linear elasticity region (under 10% strain), indicating its deformation response to external forces.

Compression tests were conducted on Ph-P and Ph-P + C-P hydrogels using CTX Texture Analyzer from Brookfield Ametek in compression mode. Disc-shaped hydrogels with a diameter of 1 cm and a height of 1 cm were prepared and placed on a flat surface. The load cell of 5 kg was used to apply the load with the speed of 0.1 mm s^−1^. Data acquisition occurred at a rate of 50 readings per second. The actual testing commenced when the load reached 0.1 N. Samples were compressed until destruction. Force-displacement graphs were plotted in triplicate for each scenario using the Texture Pro V1.0 Build 19 software.

Fiber pull-out tests were performed on the nanoplatforms of hydrogel/fiber. To prepare samples suitable for this test, fibrous mats cut in the dimensions of 5 cm by 0.5 cm were immersed in the bulk of hydrogel in a way that 3 cm of the fibers remained out of the mould. To secure the position of fibers in the middle of hydrogel, a layer of precursor solution with a volume of 3 mL was first poured into the mould, followed by 30 seconds of UV irradiation. Fibers were then placed on top of the hydrogel layer, and 3 mL more of the precursor solution was poured gently over the fibrous layer and finally irradiated by UV light for 90 seconds. 3D printing was used to fabricate a customized holder to measure the adherence of the non-woven fabric to the hydrogels. With the help of Autodesk Inventor software, a 12 × 7 mm^2^ cubic container with a height of 20 mm was designed, along with a matching lid with a 2 × 5 mm^2^ rectangular hole in the middle. The printer was a Zortrax Inventure printing with PLA polymer and standard printing parameters (*T* = 200 °C, printing speed 20 mm s^−1^). Prepared platforms were placed inside the holder and placed into the CTX Texture Analyzer from Brookfield Ametek in tensile mode. The fibers were secured within the top clamp of the tensile testing device, setting a gauge length of 10 mm. Data acquisition occurred at a rate of 50 readings per second. The actual testing commenced when the load reached 0.10 N, and the samples were stretched at a rate of 0.1 mm s^−1^ until they were pulled entirely out of the hydrogel bulk. Stress–strain and work graphs were plotted in duplicate for each condition using the Texture Pro V1.0 Build 19 software and Origin Pro.

### *In vitro* biological studies

#### Culture and seeding of L929 fibroblast cells

L929 murine fibroblast cells were cultured in DMEM supplemented with 10% FBS and 1% PS and placed in an incubator at 37 °C and 5% CO_2_. The culture medium was refreshed every two days. Cell passaging was performed when the confluence of cells reached ∼80%. For seeding, cells were detached by adding 0.05% EDTA-trypsin for 3 min and incubating the cells at 37 °C and 5% CO_2_. Subsequently, cells were collected in a Falcon tube and centrifuged at 1200 rpm for 5 min. After centrifuging, a pellet of cells was visible at the bottom of the tube. Cells were then resuspended in a 1 mL culture medium and counted. Finally, the cell suspension was further diluted in culture media to achieve a convenient cell density for seeding the samples. For the cytocompatibility indirect test, L929 fibroblast cells were seeded in 96 well tissue culture plates and incubated for 24 hours in modified DMEM at a density of 10 000 cells per well.

#### Sample sterilization and extract medium collection

Plasma-treated and untreated 14% random PCL nanofibers layered with both hydrogel types, and each side was sterilized for 30 minutes under UV light. DMEM modified with 10% FBS and 1% PS medium was added to each sample and incubated for 24 hours. The medium collected from each sample was filtered using 0.22 μm filters. Subsequently, the extracts were used to replace the culture medium in contact with the seeded cells in the tissue culture plates. The control condition was also tested by culturing the cells in a fresh medium. Cells were cultured for up to 7 days.

#### Cell viability

The viability of cells was measured by PrestoBlue assay. Cells cultured with DMEM in contact with PCL 14R/PNIPAAm hydrogel samples and fresh DMEM were treated with a solution of 10% (v/v) PrestoBlue reagent in culture medium and incubated for one hour at 37 °C and 5% CO_2_. Three replicates of each sample were analyzed at three selected time points: 1, 3, and 7 days after contact with sample extract mediums. After one hour of incubation, 100 μL aliquots of the PrestoBlue solution were transferred to a 96-well plate and analyzed at excitation 530 nm and emission at 620 nm by using a fluorometer plate reader (Fluoroskan Ascent TM Microplate Fluorometer, Thermo Scientific).

Cells were stained using a live/dead assay kit to investigate the viability of L929 fibroblasts in contact with sample extract mediums. On days 1 and 3 of culture, the samples were washed with PBS and treated with the Live/Dead staining solution composed of 0.5 μL of calcein (for staining the viable cells in green color) and 2 μL of ethidium homodimer (for red staining of dead cells) in 1 mL of PBS. Three replicates of each sample were soaked in the staining solution and incubated for 10 min at 37 °C and 5% CO_2_. Then, scaffolds were washed three times in PBS and imaged using a confocal microscope (Leica). Percentages of viable cells were counted using the Cell Counter plugin of ImageJ (National Institute of Health, USA).

Cell morphology was evaluated by a confocal microscope. Actin staining was performed on three replicates per each condition at 3 and 7 days after contact with sample extract mediums. Cell cytoskeleton and nuclei were stained by fixing the samples in 4% paraformaldehyde for 15 min at room temperature. Samples were then washed three times in PBS and treated with a solution of 0.3% (v/v) Triton X-100 for 15 min. After washing, a solution of 1% (w/v) BSA in PBS was added to the samples for 30 min. The constructs were incubated in Alexa Fluor 488 Phalloidin solution (1 : 40) and placed in the dark for 40 min. Lastly, the staining of nuclei was performed by adding 1 : 500 DAPI solution for 10 min. Samples were washed thrice in PBS and imaged with a confocal microscope (Leica).

### Statistical analysis

Data are reported in terms of mean ± standard deviation. One-way ANOVA test was assessed, and statistically significant differences are reported when *p*-value ≤0.05: *p ≤ 0.05, **p ≤ 0.01, ***p ≤ 0.001, ****p ≤ 0.0001.

## Results and discussion

### Electrospun nanofibrous layer and surface treatments

PCL stands as a prominent biomaterial frequently employed across various applications. Recognized for its biocompatibility, PCL's molecular weight exhibits significant versatility, making it adaptable for specific uses. Its semi-crystalline and hydrophobic characteristics render it ideal for applications necessitating a gradual degradation rate, complemented by its superior mechanical attributes. However, its hydrophobicity can pose challenges, leading to suboptimal wettability and hindered cell adhesion. Given the high water content of hydrogels, it becomes imperative to modify the fibers to enhance wettability in the context of hydrogel/fiber composites to improve the hydrogel-fiber bond, ensuring a cohesive and stable final product.^36^

**Fig. 1:**
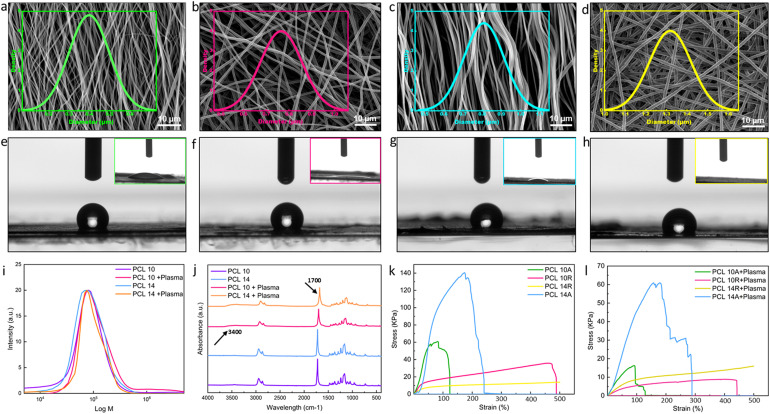
Electrospun nanofibers characteristics. SEM and fiber diameter distribution of (a) PCL 10A with average diameter of 450 nm, (b) PCL 10R with average diameter of 766 nm, (c) PCL 14A with average diameter of 809 nm, (d) PCL 14R with average diameter of 1319 nm, show in the increase of average diameter with the increase in concentration of electrospinning solution. Random fibers in each concentration are greater in size in comparison to aligned ones. Contact angle of fibers before plasma treatment and inset image of contact angle 60 minutes after plasma treatment were also reported for (e) PCL 10A, (f) PCL 10R, (g) PCL 14A, (h) PCL 14R demonstrating the hydrophilicity of nanofibers after plasma treatment, where the signs of hydrophilicity can again be seen after around 60 minutes. (i) GPC test of nanofibers before and after plasma shows no sign of destruction in the chains of PCL. (j) FT-IR of PCL10 and PCL14 before and after plasma treatment showing changes in the absorption peaks as a result of the introduction of functional groups. (k) Stress–strain graph of fibers before plasma treatment. The graph shows the enhanced yield stress for aligned fibers and increased elongation at rate for random fibers, both values elevated in case of larger fiber dimensions. (l) Stress–strain graph of fibers post plasma treatment, showing the reduced values in the mentioned conditions in comparison to pre-treatment samples.

Modulating the fiber diameter size and alignment is a viable approach to enhance the wettability of fibers and strengthen their incorporation into the hydrogel structure.^37^ Factors in electrospinning, such as solution concentration, flow rate, solvent system varieties, working distance, and applied voltage, influence fiber dimensions. Notably, solution concentration predominantly dictates fiber size.^38^ While chloroform is a popular solvent, it restricts dimensions to the microscale. We opted for adding DMF to chloroform, facilitating the generation of both nano- and micro-scale fibers.^38^ It is worth noting that there is a threshold to solution concentration to ensure the production of continuous, bead-free fibers. For this purpose, solutions of 8%, 10%, 12%, and 14% pf PCL in the same solvent system were tested, where SEM images of 8% and 12% nanofibers are shown in Fig. S1.† As it can be seen from the SEM images, 8% of nanofibers are continuous, but a lot of beads and imperfections can also be seen. By increasing the solution concentration, the fibers diameter is also increasing and beadless nanofibers are achieved. The average diameter size of 8% nanofibers was the smallest with 242 ± 43 nm, where they increased to 766 ± 92 nm for 10%, 1084 ± 140 nm for 12% and 1319 ± 102 nm for 14%. To be able to compare the effects of fiber diameter on wettability, we have selected nanofibers from 10% and 14% solutions for our study. Fig. 1a–d displays SEM images of the fibers from 10% and 14% concentration, with both random and aligned orientations, illustrating the distribution of fibers in each image. The associated data from the analysis is presented in Table 2. As evident from the SEM images and corroborated by the calculations, PCL 10A nanofibers exhibit smaller dimensions, whereas PCL 14R exhibits the biggest diameter size. As it can be seen, PCL10 nanofibers in each orientation show smaller diameters compared to nanofibers of PCL14 in the same orientation. This variation highlights the influence of solution concentration on fiber size. Aligned fibers, across both concentrations, possess reduced dimensions attributed to fiber stretching during collection for alignment. The pronounced disparity in diameter between random and aligned fibers of identical concentration is a consequence of the collector's elevated rotational speed. The correlation between fiber diameter and wettability is depicted in the contact angle visuals in Fig. 1e–h and Table 2. Larger diameter fibers with random orientation (PCL14R) appear to exhibit a tendency for hydrophilicity, potentially due to their increased surface area facilitating more interactions with water molecules. The influence of fiber alignment on dimensions and wettability can be understood by considering both the SEM visuals (Fig. 1a–d) and contact angle images (Fig. 1e–h). Contact angle measurements in Table 2 indicate a slight alteration in fiber wettability transitioning from aligned to random fibers, though the variations are minimal and may not translate to significant surface modifications.

**Table 2:** Fibers characterization before and after plasma treatment

	10A	10R	14A	14R
Fiber diameter (nm)	449 ± 100	766 ± 92	809 ± 105	1319 ± 102
CA before plasma (°)	136.9 ± 3.2	128.6 ± 2.7	131.8 ± 1.3	126.2 ± 1.7
CA after plasma *t* = 60 min	≤10	≤10	≤10	0

Plasma treatment, a recognized technique for surface activation, can be executed by exposing materials to different gases such as O_2_, N_2_, and inert gases. As a result of exposure, the covalent bonds of the surface can break, actively reacting with the functional groups. Oxygen, in particular, is a common approach to enhance interfacial adhesion in fibers, as highlighted in the literature.^39^ Good adhesion is paramount in many tissue engineering applications, as the functionality of the device is mostly dependent on the stability of the interface of the tissue under tension. This highlights the importance of stable and strong anchorage in the multi-layer structures. Due to our final goal of fabricating multi-layer nanostructures with nanofibers and hydrogels, plasma treatment was selected as one of the most efficient methods to improve the wettability of nanofibers.^40^ Plasma treatment can graft different functionalities (*e.g.*, NH_2_, OH, COOH) at the surface, making them more hydrophilic.^41^ To enhance the wettability of fibers, they were subjected to plasma treatment for a duration of 2 minutes. The chemical modification of the surface can fade quickly due to the presence of air, necessitating prompt layering. The immediate post-plasma treatment contact angle of the fibers, as presented in the insets of Fig. 1e–h and Table 2, indicates a complete shift toward hydrophilicity with no difference among the tested conditions. This change remains stable for at least 60 minutes, at which initial signs of minor hydrophobicity begin to appear in samples. One of the important matters while fabrication is to act fast enough and add the hydrogel layer in the time frame in which the functional groups are present. Upon adding the hydrogel layer with high water content, hydrogen bonds and other polar interactions with the hydrogel matrix will form. These covalent linkages, which are a result of interaction of polymer chains of the hydrogel with the functional groups of the nanofibers, are permanent and irreversible.^42^ While executing plasma treatment, it is crucial to ensure that the surface modification has not compromised the polymer chain structure. The GPC test on samples, as visualized in Fig. 1i, indicates neglectable changes in the molecular weight, suggesting the polymer chain remains almost intact. FT-IR analyses on varying PCL concentration samples pre- and post-plasma revealed peak intensity changes, signifying new functional group presence. Specific absorption peaks attributed to the formation of O–H groups are introduced to the spectrum for the samples after plasma treatment at the wavelength of 3400 cm^−1^. The peak around 1720 cm^−1^ have become broader due to the interaction of carbonyl groups with the oxygen-contained groups.^43^ All these changes showing the formation of some new bonds as a result of introduction of oxygen to the surface of the nanofibers, although there are no changes in the chemical backbone of the PCL nanofibers (Fig. 1j).

Mechanical testing on untreated and plasma-treated PCL samples assessed their Young's modulus and elongation at break. Fig. 1k presents typical stress–strain curves for pre-plasma samples. Aligned fibers, across both concentrations, exhibited superior stress and Young's modulus values (Fig. S2†), while random fibers demonstrated enhanced strain rates.

Fiber alignment greatly affects the mechanical properties of the nanofibers; this impact shows itself in the random fibers in the elevated strain rates and in aligned fibers in enhanced young modulus and yield stress. The superior strength of aligned fibers is attributed to efficient load distribution along fiber lengths, minimizing stress concentration points and breakage susceptibility.^44^ Consequently, the Young's modulus, indicative of material deformation resistance, increases. In random fibers, strain rates and the elongation at break are affected *via* alignment, as the random orientation of the fibers allows it to distribute the load unevenly between the fibers, making them more load-bearing. Also, in the case of random nanofibers, the breakage does not happen simultaneously, as fibers not in the direction of the load need to reorient for load bearing, where the voids between fibers will give them space.^45^ Fiber diameter also has an impact on the performance of the nanofibers under tensile. In both aligned and random fibers, PCL 14 shows elevated characteristics than PCL 10. In random nanofibers, PCL 14R, offers longer elongation rates than PCL 10R, where PCL 14A exhibits larger shear strength in comparison the PCL 10A. The reason lies in the fact that fibers with lower diameters tend to have more imperfections in the microstructure, which can lead to their faster breakage.^46^ Post-plasma tensile property alterations are illustrated in Fig. 1l, where all the samples show lower characteristics than pre-plasma ones. Young modulus values associated with all conditions are also reported in Fig. S2.† Young's modulus displayed reductions in all samples, likely due to the introduction of functional groups, which can weaken polymer chain intermolecular forces, resulting in diminished tensile properties.^47^

### Plasmonic hydrogel layer

Hydrogels based on acrylamide have gained significant attention in the field of biomedical applications due to their unique properties. These hydrogels are crosslinked polymeric networks that possess the ability to swell and retain a significant fraction of water within their structure without dissolving. This water retention capability arises from the hydrophilic functional groups attached to the polymeric backbone. The intrinsic resistance to dissolution is attributed to crosslinks between network chains. Such hydrogels can be responsive to external stimuli like temperature, pH, or the ionic strength of the surrounding medium, making them “smart hydrogels”. They can undergo significant volume variations in response to minor changes in environmental factors, which can be harnessed for sensing applications. One of the notable acrylamide-based hydrogels is the poly(*N*-isopropylacrylamide) or PNIPAAm-based hydrogel. When crosslinked to form hydrogels, the behaviour of these thermo-responsive polymers is significantly influenced by the volume phase transition temperature (VPTT). Above the VPTT, the polymer network shrinks, expelling the water contained within the hydrogel. The Incorporation of the plasmonic particles into the PNIPAAm-hydrogel network has been studied to evaluate potential enhancement in thermo-responsive properties of the material. The improved smart properties of this materials have been used in many applications, such as biosensing applications^16^ and photothermal therapy.^48^

**Fig. 2:**
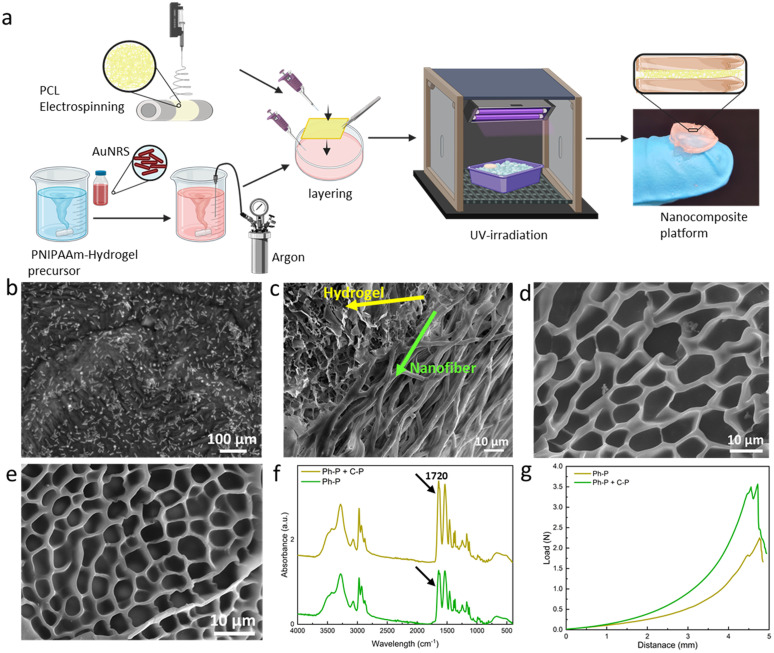
Fabrication steps of the platform and hydrogel characterization. (a) Scheme of the fabrication of the layered nanoplatform. Briefly, the fabrication steps provide the addition of gold nanorods to the hydrogel precursor solution, argon bubbling prior to layering, addition of treated/untreated PCL nanofibers and crosslinking *via* UV-irradiation. (b) TEM micrograph of AuNRs in the hydrogel matrix. (c) Cross-section FE-SEM image of the platform, showing the incorporation of fibers into the hydrogel network. (d) FE-SEM images of the Ph-P hydrogel (e) FE-SEM of Ph-P + C-P hydrogel showing smaller pores. (f) FT-IR spectra of both hydrogels, confirming the increased crosslinking density of Ph-P + C-P hydrogel. (g) Force-displacement curves of hydrogels, showing enhanced toughness of the Ph-P + C-P hydrogel in comparison to C-P hydrogel.

Fig. 2a illustrates the fabrication methodology of the fiber/hydrogel nanoplatform using a layering technique. The hydrogel precursor solution was prepared and stirred overnight before the integration of gold nanorods. Prior to layering, it is required to bubble the precursor solution with argon to eliminate oxygen. This step is needed due to the chain reaction for polymerization which can be induced *via* Irgacure as photoinitiator. Optionally, APS (as chemical initiator) and TEMED (as catalyst) can be introduced to increase the hydrogel's crosslinking density. APS, upon heating, liberates sulfate radicals capable of breaking acrylamide's double bonds, thus creating crosslinking sites. Concurrently, TEMED accelerates radical formation even at reduced temperatures. Subsequently, untreated/treated PCL nanofibers are positioned within a specified mould over which the precursor solution is poured. The mould is then cooled in an ice bath to regulate temperature during UV exposure, and the full hydrogel crosslinking is achieved within 90 seconds of UV irradiation. The composite's final architecture is visually represented in the accompanying schematic (Fig. 2a).

Gold nanorods' incorporation within the hydrogel matrix is validated through FE-SEM analysis, as depicted in Fig. 2b. The nanorods exhibit an average length of 55 ± 18 nm and a thickness of 15 ± 5 nm. Fig. 2c presents FE-SEM images of the composite's cross-section, revealing a stratified structure with seamless integration of fibers within the hydrogel matrix. FE-SEM images of the layered platform, showing the nanofibrous layer between two layers of hydrogel is shown in Fig. S3.† Evidently, the precursor solution permeates the fiber layers, resulting in a sturdy cross-section post-crosslinking. Fig. 2d shows a hydrogel formed solely through photo-polymerization (Ph-P) with an average pore size of 11.05 ± 1.96 μm. In contrast, Fig. 2e highlights a hydrogel synthesized using photo-polymerization and chemical polymerization (Ph-P + C-P) with APS and TEMED where the pore sizes have an average dimension of 5.16 ± 0.68 μm. The influence of APS and TEMED on hydrogels' degree of polymerization is discernible in these FE-SEM images. Higher crosslinking density manifests as reduced pore sizes, leading to a more rigid network.^49^ This densification is further corroborated by FT-IR analysis of lyophilized samples, as shown in Fig. 2f. Peaks at 2900 and 2740 cm^−1^ correspond to asymmetrical and symmetrical C–H stretching, respectively, while the peak at 1720 cm^−1^ is attributed to the C=O stretching vibration of the carboxyl group in acrylic acid. The pronounced peak intensity at 1720 cm^−1^ is indicative of elevated crosslinking. Mechanical properties, assessed through compression tests on hydrogels with varying crosslinking densities, are depicted in Fig. 2g. The curve's gradient, representing the elastic modulus, is steeper for hydrogels synthesized using Ph-P + C-P. Both the yield and ultimate strengths are markedly higher for Ph-P + C-P.

### Mechanical pull-out test

A customized mechanical test, known as the fiber pull-out test, was devised to assess the adhesion strength between a fiber bundle (acting as the reinforcing material) and the encompassing matrix material, such as a hydrogel. This test serves as a valuable tool for evaluating the interfacial bonding within the layers. The test procedure involves partially embedding a filament or bundle of fibers within the hydrogel matrix and subsequently applying a force to extract the fiber from the matrix.^50^ The fiber pull-out test offers critical information into the strength and integrity of the fiber–matrix interface and, by extension, the overall performance of the composite material. The level of adhesion between these two components provides a practical indicator of the composite's load-bearing capacity and deformation resistance, reflecting the effectiveness of their integration.^51^

**Fig. 3:**
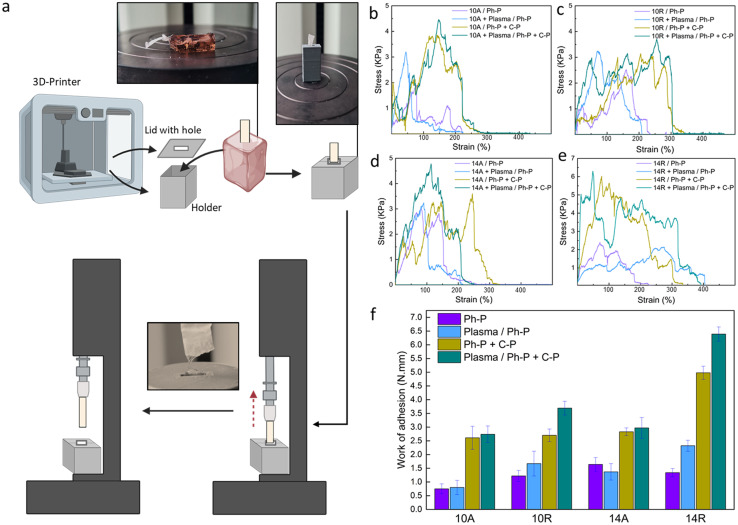
Fiber pull-out test. (a) Scheme of the customized 3D-printed holder for the nanoplatforms and the test steps. (b–e) Stress–strain curves for (b) PCL10A, (c) PCL10R, (d) PCL14A, (e) PCL14R paired with hydrogel in all the conditions. (f) Nanoplatforms' toughness, measured by calculating the work per unit volume for each group set as an indicative value of the adhesion and incorporation of interfaces.

To facilitate this test, a custom-made setup was designed, as shown in Fig. 3a. Overcoming one of the primary challenges of this test, such as securing the hydrogel within the tensile machine, required innovative approach. To achieve this, a 3D-printed holder was designed to distribute the pressure exerted by the tensile grips without imposing stress on the hydrogel. Additionally, the inherent tendency of the hydrogel to displace along with the embedded fibers under tension necessitated to address a few more requirements. A lid was created for the holder, featuring a hole specifically designed to the size of the fiber, as can be seen in Fig. 3a. The holder was then fixed into the lower grip of the tensile machine while the upper grip securely held the fiber bundle. The test proceeded until a complete separation of the two materials was achieved. Fig. 3b–e shows the stress–strain curves for all the samples in different conditions, where Fig. 3f shows the work of adhesion, needed for the fiber pull-out for each sample and group. Parameters which can affect this investigation are the fiber dimension, alignment and surface treatment, and toughness of hydrogel. With a quick glance, it can be stated that samples featuring Ph-P + C-P hydrogel, which is a stiffer hydrogel compared to Ph-P hydrogel, exhibit a higher work of adhesion. The results indicate that the force needed to pull the fibers is not solely the force for separating fibers from the hydrogel but is predominantly associated with the force required to fracture the hydrogel network in close proximity to the interface. This observation is supported by the fact that the fiber bundles extracted from the hydrogel matrix consistently, retain traces of hydrogel on their surfaces, showing the robust integration of fibers into the hydrogel network (Fig. S4†).

While plasma treatment enhanced the wettability of the PCL nanofiber surfaces, promoting an expected increased intra-layer adhesion based on the previous results, the impact of plasma treatment in the fiber pull-out test is much less than the hydrogel part. Samples with plasma treatment in each group of aligned nanofibers show very small increase compared to the not treated ones. This difference is shown to be much more in the case of samples with randomly oriented nanofibers. This can be because of the more exposed cites on random fibers in the procedure of plasma treatment.^52^ As discussed previously, fiber diameter and orientation, as other parameters, have some effects on the adhesion of nanocomposite. PCL 14R, with the most larger diameter size, has the greatest work of adhesion, whereas PCL 10A has the lowest values in pre- and post-treated samples. As mentioned before, PCL14, with a bigger fiber diameter, offers more strong fibers and randomly aligned nanofibers, offer more void and space for the precursor solution to penetrate, which can provide a more robust interface.

In summary, the mechanical response of PCL nanofibers within the composite system is profoundly influenced by the crosslink density and toughness of the hydrogel in all situations. Fiber diameter and orientation also play important roles as they will offer more space for penetration of the hydrogel layer. Finally, the plasma treatment, especially in randomly aligned nanofibers, can influence the required force and, hence the work of adhesion. The fiber pull-out test reveals that the force required for fiber extraction is intrinsically tied to the resilience of the hydrogel network near the interface, highlighting the importance of this interfacial region in composite materials.

### Biocompatibility of nanoplatforms

To evaluate the biological properties of the nanoplatforms and verify their potential for biomedical applications, the material interaction with L929 fibroblast cells was investigated.^53^ According to the mechanical tests, PCL14R nanofibers showed the most robust interface and hence were selected as a representative to assess the biocompatibility of nanoplatforms.

**Fig. 4:**
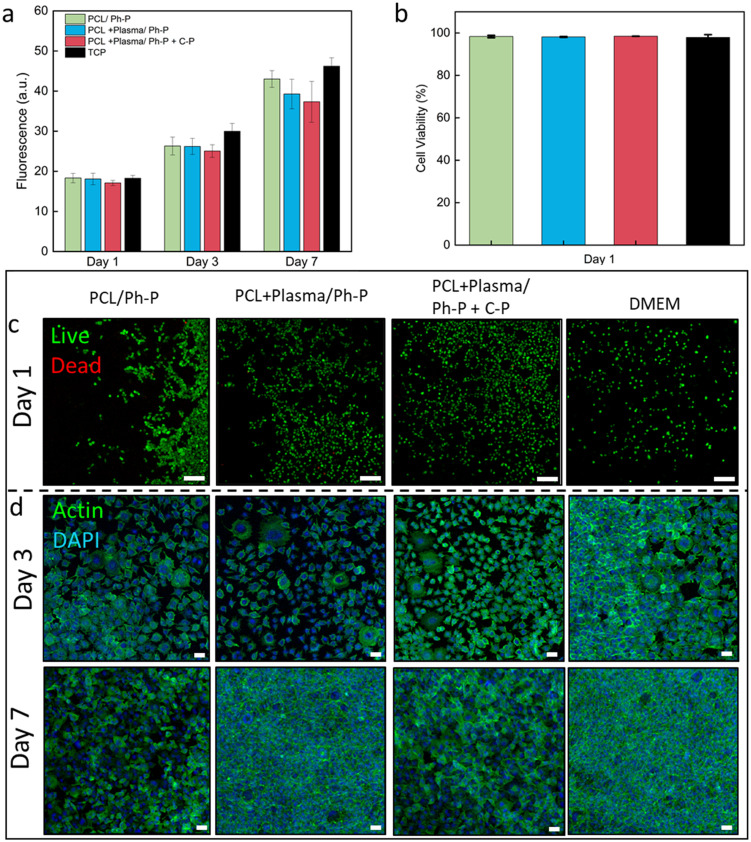
*In vitro* biological response of L929 fibroblasts seeded on TCP and cultured with extracted medium of PCL14R nanofibers/hydrogel in different conditions. (a) Increasing trend of cell viability up to 7 days of culture tested. (b) Percentage of cell viability calculated from live and dead images, showing ≥97% cell viability at day 1 of culture. (c) Live and dead images showing live cells (green) and dead cells (red) at day 1 of the culture. Scale bar: 50 μm. (d) Confocal images of samples stained with Actin (green) and DAPI (blue), visualizing cell cytoskeleton and nuclei, respectively. Scale bar: 50 μm.

The viability of L929 cells seeded on TCP and cultured using extracted medium was evaluated and compared to control samples cultured with fresh medium. Linear cell growth is reported in Fig. 4a, showing increasing signals at each time point (1, 3, and 7 days) for all conditions. No significant difference between the conditions was measured at any time, confirming the indirect cytocompatibility of nanoplatforms, either with plasma-treated nanofibers or having additional C-P reagents for denser crosslinking.

Utilizing a Live/Dead assay kit, cells seeded on the culture plate were stained, with live cells appearing green and dead cells showing in red. This observation shows the predominant presence of viable green cells cultured in the presence of sample extracts, as shown in Fig. 4b and Table S1,† affirming the employed materials' indirect cytocompatibility. Illustrative Live/Dead images from day 1 and day 3 of culture are reported in Fig. 4c and S5,† respectively. Across the evaluated conditions, no significant variations were identified at any given time interval.

Confocal microscopy images of samples stained with Actin/DAPI showed the cell cytoskeleton and nuclei after 3 and 7 days of culture (Fig. 4d). The images allow the visualization of the elongated morphology of L929 fibroblasts, exhibiting a characteristic spindle shape from the early phase and evident cell proliferation and population at the later stage of culture, with no significant difference between conditions.

## Conclusions

In this work, we investigated factors that could potentially result in a more robust interface between the plasmonic hydrogel layer and electrospun nanofibers in multi-layer nanocomposites. The nanofibrous mats of PCL were successfully fabricated from 10% and 14% solutions, offering nanofibers with an average diameter of below and above 1 μm, respectively, in random and aligned orientations. O_2_ plasma treatment was applied to the nanofibers' surface to enhance the materials' hydrophilicity. Morphological and mechanical investigations showed each parameter's impact on the nanofibers' wettability and hydrophilicity, which can influence their interface for further compositing steps. Fiber diameter and orientation have a neglectable effect on the nanofibers' hydrophilicity while applying plasma treatment makes them completely wettable for at least 60 min. Orientation and fiber diameter influence the mechanical tests, as random nanofibers offer more elongation at break in tensile and aligned fibers can bear higher shear stress. These mechanical values were significantly decreased post-plasma treatment due to the presence of new functional groups and reduced intramolecular forces. AuNRs were added to PNIPAAm hydrogel precursors with Ph-P (Irgacure as a photoinitiator) and Ph-P + C-P (Irgacure as photoinitiator and APS and TEMED as chemical initiator and catalyst) polymerization routes, then layered to nanofibrous layer to achieve a multi-layer nanocomposite. Morphological illustrations confirm the presence and distribution of AuNRs in the hydrogel layer. FE-SEM images and mechanical compression tests proved the increased crosslinking density in the Ph-P + C-P hydrogel due to smaller pore sizes and higher yield and toughness. A mechanical pull-out test was performed on all the samples, as the adhesion and robustness of the interface are crucial in layered smart platforms to provide stable construct within all changes and alterations. For this purpose, a custom-made 3D holder for the samples was printed, and the work of adhesion was calculated for all the sample sets. Nanofibers with larger diameters and random orientation had a more considerable work of adhesion, and this work was reduced by decreasing the fiber diameter and aligning. The impact of plasma treatment for aligned fibers was neglectable, whereas in random fibers, there is a noticeable increase in the adhesion of layers. The most significant alterations were noticed with the increase of the crosslinking density of the hydrogel, as the values increased for all the sample sets. It can be concluded that the impact of hydrogel stiffness is more significant, making it visible that the adhesion energy is mainly attributed to the force to break the hydrogel network near the fiber interface. Finally, cytocompatibility indirect tests with L929 fibroblasts show cell viability and proliferation in all conditions, proving that the nanoplatforms can be an ideal candidate for biomedical applications.

## Author contributions

F. P. conceived the idea. Y. Z. designed the experiments, fabricated the nanoplatforms. Y. Z. performed the morphological characterization of nanofibers. M. L tested the GPC of nanofibers before and after treatment. Y. Z. and A. Z performed the morphological characterization of hydrogels. Y. Z and S. S. Z tested mechanical pull-out experiments. P. N. printed a 3D holder for the mechanical tests. Y. Z., C. R. and A. K. K conducted cell studies. Y. Z. wrote the manuscript. All authors discussed the results and commented on the manuscript. F. P. supervised the project.

## Conflicts of interest

There are no conflicts to declare.

## Supplementary Material

NA-006-D3NA01022H-s001
