# Supplementary material for: Developing strategies to optimize the anchorage between electrospun nanofibers and hydrogels for multi-layered plasmonic biomaterials
Source: Nanoscale Adv. 2024 Jan 30;6(4):1246–58. doi: 10.1039/d3na01022h (PMC10863722; doi:10.1039/d3na01022h)
Supplement: NA-006-D3NA01022H-s001 [file NA-006-D3NA01022H-s001.pdf]

Electronic Supplementary Material (ESI) for Nanoscale Advances.
This journal is © The Royal Society of Chemistry 2024

ARTICLE

Developing strategies to optimize the anchorage between electrospun nanofibers and hydrogel for multi-layered plasmonic biomaterials

Yasamin Ziai,[a] Massimiliano Lanzi [b] ,Chiara Rinoldi [a] , Seyed Shahrooz Zargarian [a] ,Anna Zakrzewska [a], Alicja Kosik-Kozioł [a], Paweł Nakielski [a], and Filippo Pierini*[a]

Supplementary Information

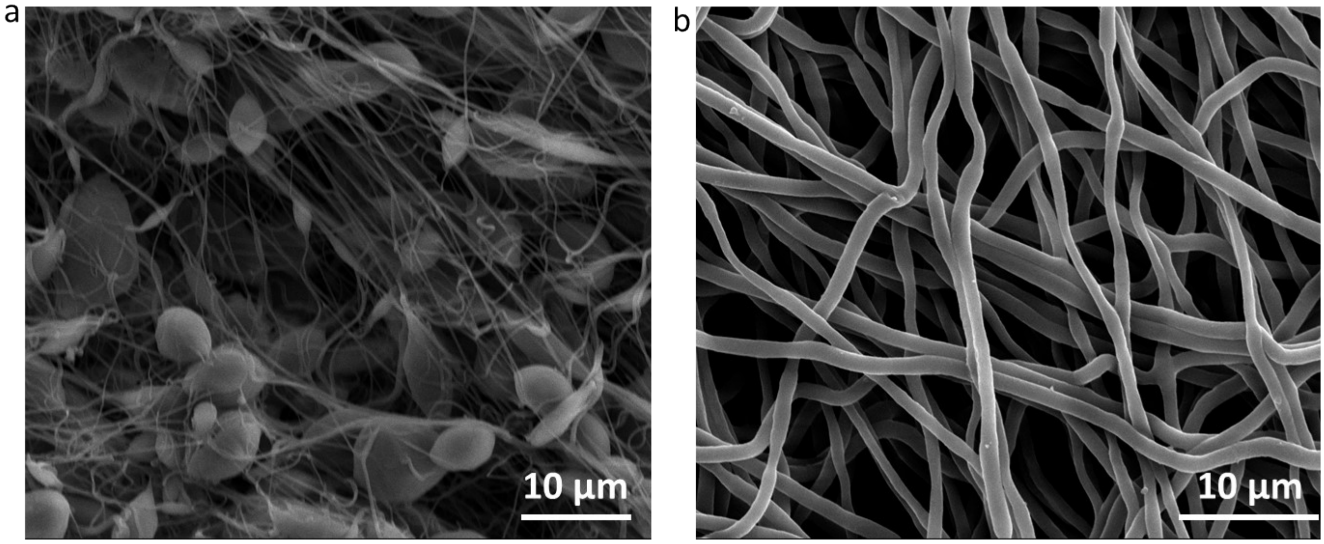

Figure S1. SEM images of PCL nanofiber. a) 8% concentration with an average diameter of 242 ± 43 nm, beads in the nanofibers, b) 12% concentration with an average diameter of 1084±0.14 nm, continuous nanofibers.

Please do not adjust margins

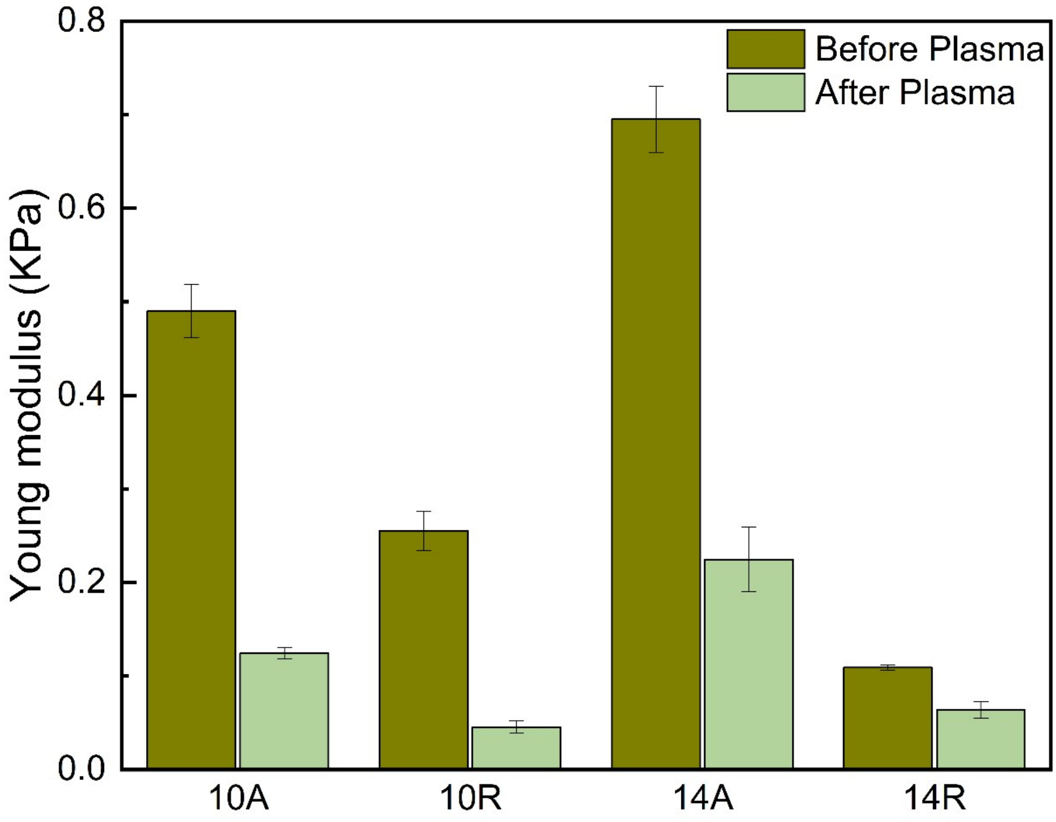

Figure S2. Young modulus of PCL nanofibers before and after plasma treatment

This journal is © The Royal Society of Chemistry 20xx

Please do not adjust margins

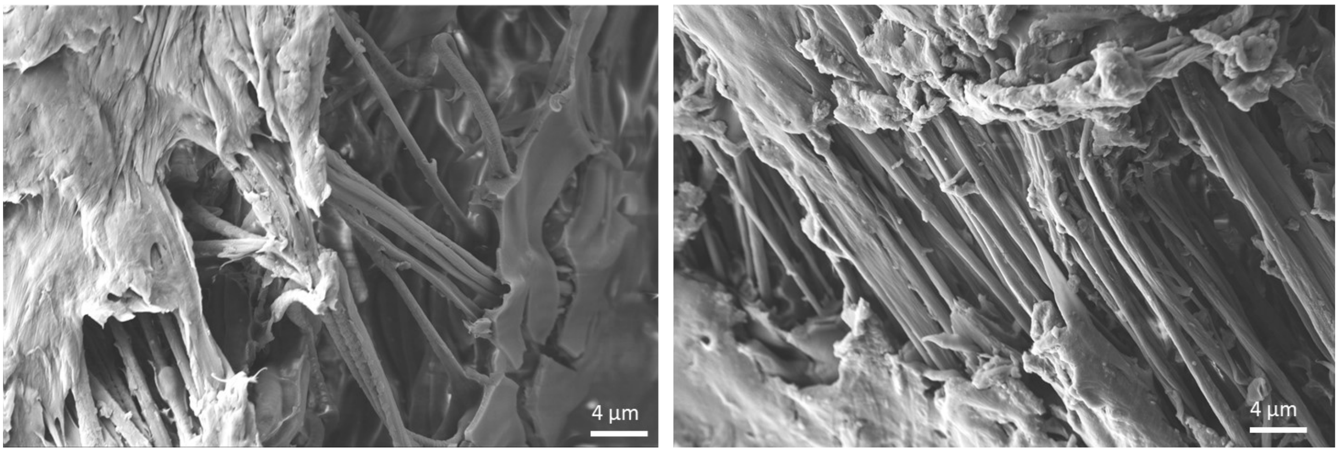

Figure S3. FE-SEM images of nanocomposite cross-section, showing the layered structure of the platform, with a layer of nanofibers between two layers of hydrogel.

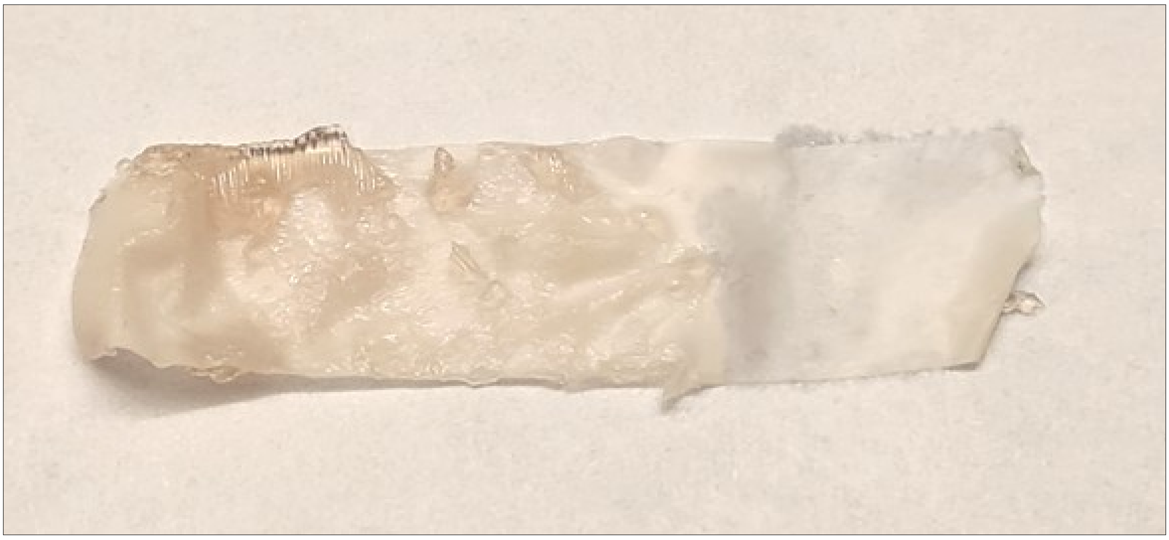

Figure S4. Image of the fiber bundle after pull-out test. The traces of the hydrogel remaining on the surface of the fibers, indicate the stable interface between the layers of the nanocomposite. This image also confirms the fact that the force required for the pill-out test is mostly spent on the destruction of the hydrogel network very close to the interface with the fibrous layer.

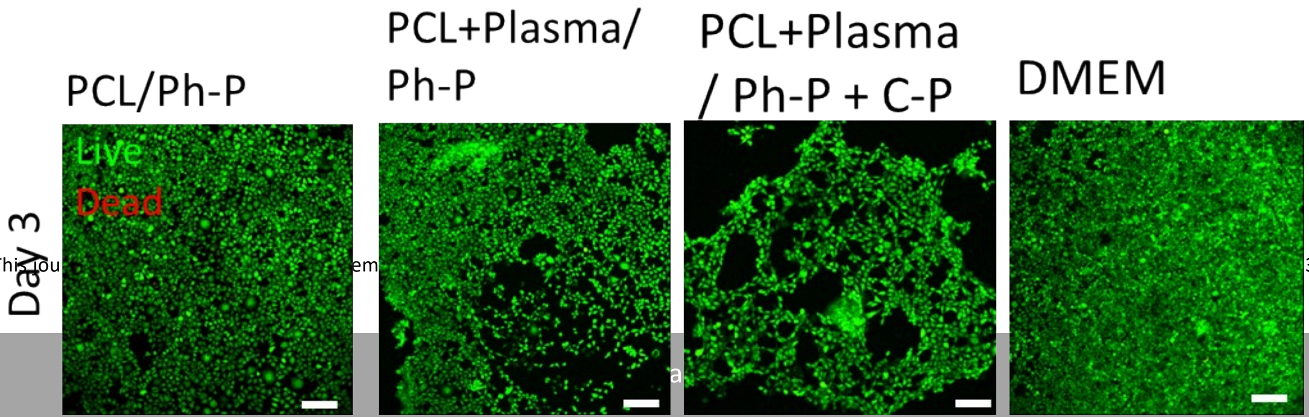

Please do not adjust margins

Figure S5. *In vitro* biological response of L929 fibroblasts seeded on TCP and cultured with the extracted medium of PCL14R nanofibers/hydrogel platforms in the form of Live and dead images. Live cells (green) and dead cells (red) on day 3 of the culture. Scale bars: 50µm

Samples	**Cell viability (%)**
**PCL/Ph-P**	97.96
**PCL+Plasma/Ph-P**	94.02
**PCL+Plasma/ Ph-P + C-P**	94.84
**DMEM**	98.45

Table S1. *In vitro* biological response of L929 fibroblasts seeded on TCP and cultured with the extracted medium of PCL14R nanofibers/hydrogel platforms in the form of Live and dead image analysis on day 3 of culture.

This journal is © The Royal Society of Chemistry 20xx

Please do not adjust margins
